# Cross-omics strategies and personalised options for lung cancer immunotherapy

**DOI:** 10.3389/fimmu.2024.1471409

**Published:** 2024-09-25

**Authors:** Yalan Yan, Siyi Shen, Jiamin Li, Lanqian Su, Binbin Wang, Jinghan Zhang, Jiaan Lu, Huiyan Luo, Ping Han, Ke Xu, Xiang Shen, Shangke Huang

**Affiliations:** ^1^ School of Clinical Medicine, The Affiliated Hospital, Southwest Medical University, Luzhou, China; ^2^ Intensive Care Unit, Xichong People’s Hospital, Nanchong, China; ^3^ Department of Anaesthesiology, Southwest Medical University, Luzhou, China; ^4^ Department of Oncology, Chongqing General Hospital, Chongqing University, Chongqing, China; ^5^ Department of Respiratory and Critical Care Medicine, The Affiliated Hospital, Southwest Medical University, Luzhou, China; ^6^ Department of Oncology, The Affiliated Hospital, Southwest Medical University, Luzhou, China

**Keywords:** lung cancer, immunotherapy, precision medicine, multi-omics, individualised therapy, immune checkpoints

## Abstract

Lung cancer is one of the most common malignant tumours worldwide and its high mortality rate makes it a leading cause of cancer-related deaths. To address this daunting challenge, we need a comprehensive understanding of the pathogenesis and progression of lung cancer in order to adopt more effective therapeutic strategies. In this regard, integrating multi-omics data of the lung provides a highly promising avenue. Multi-omics approaches such as genomics, transcriptomics, proteomics, and metabolomics have become key tools in the study of lung cancer. The application of these methods not only helps to resolve the immunotherapeutic mechanisms of lung cancer, but also provides a theoretical basis for the development of personalised treatment plans. By integrating multi-omics, we have gained a more comprehensive understanding of the process of lung cancer development and progression, and discovered potential immunotherapy targets. This review summarises the studies on multi-omics and immunology in lung cancer, and explores the application of these studies in early diagnosis, treatment selection and prognostic assessment of lung cancer, with the aim of providing more personalised and effective treatment options for lung cancer patients.

## 1 Introduction

Lung cancer has been one of the most common malignant tumours globally over the past decades. Despite the widespread use of conventional treatments such as surgery, radiotherapy, chemotherapy and targeted drug therapy, the five-year survival rate for lung cancer is usually less than 20% (1). Additionally, at all stages, less than 7% of patients survive more than ten years after diagnosis (2). In recent years, the emergence of immunotherapy has marked a revolution in cancer treatments, which not only has an acceptable safety profile, but also produces durable therapeutic responses in a wide range of patient populations (3). Nonetheless, lung cancer exhibits significant histological heterogeneity, diverse genomic profiles, and differential responses to therapy (4), and still poses significant challenges for immunotherapy and prevention.

With the rapid development of multi-omics technology, covering genomics, transcriptomics, proteomics and metabolomics, our understanding of lung cancer is deepening (5, 6). Multi-omics technology has constructed a progressive analysis framework from the genetic basis to the effect of environmental exposure (7), and has deeply analysed the pathogenesis, pathophysiological process and molecular basis of immunotherapy of lung cancer, which has provided a strong support for the scientific formulation of precise treatment strategies.

The aim of this review is to explore recent advances in multi-omics studies of lung cancer and their potential applications in early diagnosis, treatment selection and prognostic assessment. By integrating immunotherapy and multi-omics data in order to better understand the complex molecular network of lung cancer, it provides new ideas and methods for individualised treatment and precision medicine of lung cancer.

## 2 Lung cancer immunotherapy and genomics

Lung cancer, as a highly heterogeneous disease, has been profoundly influenced by molecular biology in its pathogenesis and therapeutic strategies. In non-small cell lung cancer (NSCLC) and small cell lung cancer (SCLC), unique molecular features of different histological subtypes have been revealed through the identification of specific genetic variants and epigenetic modifications, thus providing new directions for individualised treatment of lung cancer.

**Figure 1:**
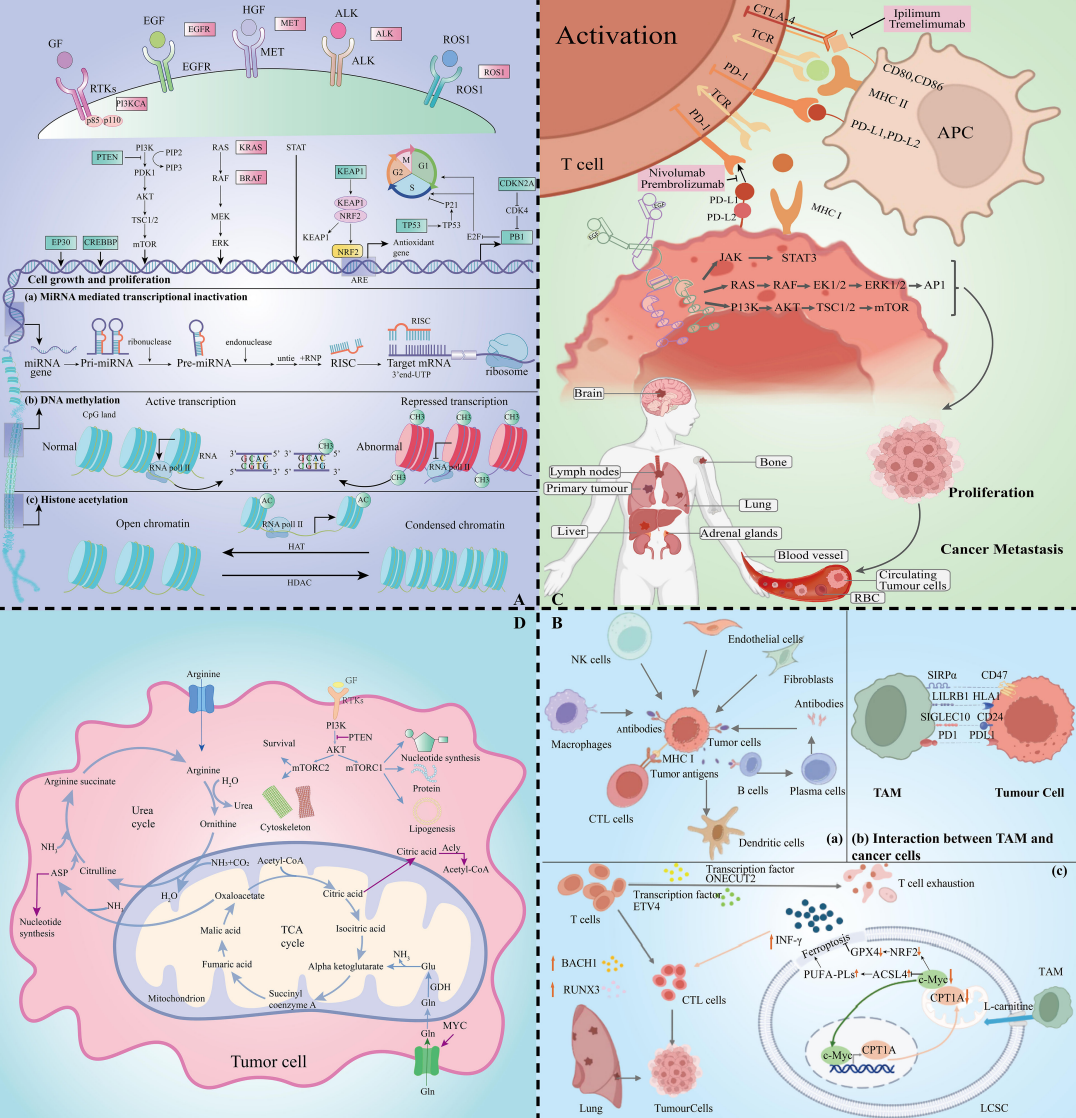
Lung cancer multidimensional histologic analysis map. **(A)** Exploratory mapping of changes in metabolomic profiles in lung cancer pathogenesis. **(B)** Immune cell profiles revealed by transcriptomics in the tumour microenvironment. **(C)** Critical mapping of proteomic changes during lung cancer progression. **(D)** Resolution of metabolic profiles in lung cancer.

In NSCLC, histological subtypes frequently dominated by lung adenocarcinoma (LUAD) and squamous cell carcinoma are common (8). The complexity of NSCLC is reflected in its variable genetic variants. Common target gene driver mutations include genes such as epidermal growth factor receptor (EGFR), KRAS, MET, BRAF, ALK, ROS proto-oncogene 1 (ROS1) and RET (9) (**Figure 1A**). Through combined whole exome sequencing (WES) technology, it was found that common mutations in LUAD include tumour suppressor genes TP53 (46%), STK11 (17%), KEAP1 (17%), NF1 (11%), RB1 (4%) and CDKN2A (4%), as well as chromatin modification genes SETD2 (9%), ARID1A (7%), SMARCA4 (6%) and RNA splicing genes RBM10 (8%) and U2AF1 (3%) (10). Mutations in the genes FGFR1, NRF2, AKT1 and DDR2 are particularly prominent in lung squamous cell carcinoma (10).

For SCLC, deep sequencing of key oncogenes by advanced integrated mutational profiling (MSK-IMPACT) technology (11) revealed inactivating mutations or deletion of tumour suppressor genes such as TP53, RB1, KMT2D, PTEN, NOTCH1, CREBBP, FAT1, NF1 and APC, and inactivating mutations in PIK3A, EGFR and KRAS activating mutations in oncogenes (12). Unlike NSCLC, SCLC is often accompanied by the expression of MYC oncogenes, which contribute to rapid cell proliferation and lead to DNA replication stress (13). In addition, epigenetic modifications play a key role in lung carcinogenesis, and heritable chromatin modifications such as DNA methylation, histone modifications, and non-coding RNA regulation regulate gene expression without altering the DNA sequence (9, 10, 14–16) (**Figure 1A**). Epigenetic mutations and disruptions are strongly associated with multiple tumour types, providing new ideas for targeted lung cancer therapy based on molecular subtype differences.

Genomics plays an important role in the classification, treatment and prognostic assessment of lung cancer. Traditionally, lung cancer classification was based on histological patterns, whereas advances in genomics have allowed lung cancer to be characterised also by tumour biomarkers and genetic alterations. For example, Stephen J Murphy et al. defined a common origin or lineage of lung cancer by analysing genomic rearrangements and somatic DNA linkages, and used these specific DNA linkages as precise tumour markers to differentiate between primary and metastatic lung cancer (17).

Genome sequencing technology has revealed key genetic variants in lung cancer, facilitating the development of personalised treatment strategies. The study noted that in non-small cell lung cancer (NSCLC), aberrant activation of the PI3K-AKT-mTOR pathway is closely associated with resistance to EGFR tyrosine kinase inhibitors (EGFR-TKIs), and that its activation is mainly caused by PIK3CA, AKT1 mutations and PTEN deletion. This discovery led to the development of drugs targeting mTOR (e.g., everolimus and temsirolimus) and EGFR-TKIs targeting EGFR and ALK (e.g., ositinib, gefitinib, ceritinib, and loratinib), which have demonstrated clinical efficacy in the treatment of lung cancer (18) (**Table 1**).

**Table 1:** Targeted therapeutics drugs and targets in a genomic perspective.

Modifiable targets	Therapeutic drug	Reference
EGFR	Osimertinib	(19)
	Gefitinib	(20)
	Dacomitinib	(21)
	Erlotinib	(22)
	Afatinib	(23)
	Amivantamab	(24)
ALK	Crizotinib	(25)
	Ceritinib	(26)
	Alectinib	(27)
	Brigatinib	(28)
	Lorlatinib	(29)
RET	Cabozantinib	(30)
	Selpercatinib	(31)
	Pralsetinib	(32)
ROS1	Crizotinib	(33)
	Entrectinib	(34)
	Lorlatinib	(35)
MET	Glesatinib	(36)
BRAF V600E	Dabrafenib	(37)
	Trametinib	(38)
	Vemurafenib	(39)
KRAS	Adagrasib	(40)
	Sotorasib (AMG 510)	(41)
VEGFR	Bevacizumab	(42)
	Ramucirumab	(43)

Immune checkpoint inhibitors (ICIs) have become key agents in tumour immunotherapy, especially in the treatment of lung cancer. However, their potential to cause immune-related adverse effects makes the search for biomarkers that predict response to ICI therapy crucial (9). Investigators assessed early predictors of anti-PD-L1 therapy by analysing circulating tumour DNA (ctDNA), and their study showed that a reduction in the frequency of the variant allele was associated with tumour shrinkage after 6 weeks of treatment, providing a valuable non-invasive method for predicting the effectiveness of treatment (44).

## 3 Lung cancer immunotherapy and transcriptomics

In the field of tumour immunotherapy, targeting the tumour microenvironment (TME) for precision medicine is one of the latest research directions. In this process, immune cells play a key role (45). Immune cell interactions are regulated by transcription factors and further contribute to the immune response. Wu et al. identified interactions between cancer cells and endothelial cells, fibroblasts, and macrophages by single-cell RNA sequencing analysis of NSCLC patient samples, revealing multiple signalling pathways (e.g., EGFR, NOTCH, WNT, and PDGF, etc.) that are associated with carcinogenesis (**Figure 1B**). These findings shed more light on the molecular interactions and immunoregulatory mechanisms of NSCLC and provide a new perspective on the treatment of lung cancer (46).

In TME, individual cells are precisely regulated by transcription factors. Through the regulation of transcription factors, the killing of cells can be modulated. ONECUT2 and ETV4 were found to be likely potential regulators of CD8 T cell depletion in the blood of NSCLC patients, whereas the transcription factors BACH1 and RUNX3 were up-regulated in CD8 T cytotoxic subpopulations. Thus, regulation of these transcription factors may drive cytotoxic immune responses in NSCLC (47). Immune cell macrophages (TAM) in TME are among the most common immunosuppressive cells. Increased TAM in TME has been associated with immunotherapy resistance by transcriptomic analysis, and its expression is regulated by genes such as CD27, ITGAM and CCL5 (48). Recent studies have shown that TAM interacts with carnitine palmitoyltransferase 1A (CPT1A), increasing resistance to iron death and inactivation of CD8 T cells in lung cancer. Therefore, the use of CPT1 inhibitors enhances the killing of tumour cells by chemotherapy or immunotherapy (49) (**Figure 1B**).

In the field of lung cancer treatment, transcriptomics is often combined with other histological approaches to extend its application. By combining transcriptomics and metabolomics, the researchers analysed the effects of AZD-6482 (a PI3Kβ-targeted inhibitor) on 28 metabolite-related genes in LUAD. They found that the expression of three genes, LDHA, PPAT, and SMS, was increased in untreated LUAD samples; whereas after treatment with AZD-6482, the expression of these genes was significantly decreased, suggesting that the inhibitor may improve the prognosis of LUAD patients (50). By joining forces with proteomics, Qing et al. used Cancer Cell Line Encyclopaedia (CCLE) RNA sequencing and proteomics profiles in human NSCLC cell lines to identify genes that are pan-sensitive and pan-resistant to drugs used in the treatment of NSCLC with systemic or targeted therapies (51).

## 4 Lung cancer immunotherapy and proteomics

With the rapid development of mass spectrometry (MS) technology, large-scale protein analysis has become a hotspot in scientific research, in which proteomics has achieved remarkable results in the study of protein phosphorylation, interaction, structure and function (52–55). In particular, proteomics has shown great potential for the discovery of new therapies and biomarkers. These biomarkers come from a wide range of sources, including body fluids and specific samples from lung cancer studies, such as breath condensate (56–59).

In the study of lung cancer proteomics, proteins in receptor tyrosine kinases such as EGFR and ALK and their downstream signalling pathways play a key role in the pathological process of lung cancer (60–62). EGFR, as a member of the ErbB family, promotes malignant cell survival, proliferation, etc. through a series of biochemical processes, making EGFR and its downstream signalling pathway an important target for lung cancer therapy.

In addition, the discovery of immune checkpoints (ICP) has led to a major breakthrough in the field of immunotherapy (63). In normal physiology, ICP maintains immune system homeostasis, but tumour cells evade immune attack by expressing ICP proteins (3). Among them, the interaction of programmed death receptor 1 (PD-1) with programmed cell death ligand-1 (PD-L1), cytotoxic T-lymphocyte-associated protein-4 (CTLA-4) and CD80/86 is the main mechanism of tumour cell escape (**Figure 1C**). Significant progress has been made in the development of targeted therapeutic agents for lung cancer against these immune checkpoint proteins (64). By blocking the function of these proteins, the immune system in the patient’s body is activated to recognize and attack tumour cells more effectively, bringing new therapeutic hope to lung cancer patients. Over the past decade, tyrosine kinase inhibitors (TKIs) have made significant advances in the treatment of cancer, especially NSCLC.EGFR-TKI, as a potent agent for the treatment of over-activation of EGFR signalling, has been developed for multiple generations with remarkable efficacy (54, 65).

In the development of the field of immunotherapy, it is particularly important to achieve selective destruction of tumours by activating the immune response of T cells (64, 65). PD-1/PD-L1 inhibitors in combination with chemotherapy have become the standard of care in advanced NSCLC (62, 66). In clinical study finds,PD-1 antibodies such as Nivolumab and Pembrolizumab demonstrate durable efficacy in a variety of cancers (67, 68) (**Figure 1C**). In addition, anti-CTLA-4 antibodies such as Lpilimumab and Tremelimumab play an important role in immunotherapy.

Immunotherapy has great potential in the field of cancer treatment, including checkpoint inhibitors, monoclonal antibodies, and over-the-counter cell transplantation (69). Scholars such as Wang and Chiu emphasized that the combination of multiple therapies is the key to enhancing the effectiveness of cancer treatment and is expected to significantly improve patient survival rates (70, 71).

## 5 Lung cancer immunotherapy and metabolomics

Metabolomics delves into metabolite changes in organisms, providing new insights into the pathology and drug mechanisms of lung cancer. By analysing lung cancer samples and identifying metabolic markers closely related to lung cancer, it brings new perspectives for early diagnosis, treatment planning and prognosis assessment (72–74).

In the oncogenic transformation of lung cancer cells, there are significant metabolic changes that are particularly dependent on energy sources such as ATP. Among these, the Warburg effect is particularly pronounced in lung cancer cells, where their metabolic needs are met by increased glucose uptake and support nucleotide and amino acid biosynthesis (75). SCLC is significantly dependent on exogenous arginine, which is associated with the deficiency or low expression of arginine succinyl synthase 1 (ASS1) (76, 77). The study found that up to 45% of SCLC samples and 50% of cell lines exhibited ASS1-negativity, highlighting the importance of arginine biosynthesis downregulation in the progression of lung carcinogenesis (78, 79).

The interaction between signalling and metabolism is critical in lung cancer research. mTOR kinases in the PI3K/Akt/mTOR pathway form the mTORC1 and mTORC2 complex, which influences protein, nucleotide, and lipid metabolism (80) (**Figure 1D**). MYC gene changes affect bioenergetic processes (81). Changes in metabolic state can also inversely regulate signalling pathway activity, e.g., mTORC1 activity is reduced during energy shortage (82).

Developing more effective lung cancer treatment regimens by modulating metabolic pathways or monitoring the disease using metabolic markers. In particular, lung cancer immunotherapy is closely linked to metabolomics, an important cornerstone of lung cancer treatment (83). The markers provided by metabolomics support personalized strategies for immunotherapy and improve treatment efficiency. In addition, the study by Ma et al. revealed the relationship between the regulation of amino acid metabolism, hypoxia-inducible factor-1 (HIF-1) and PI3K-Akt pathways and ositinib resistance, providing new perspectives for understanding the mechanism of drug resistance (84). These studies emphasize the critical role of metabolomics in monitoring marker changes after treatment (85).

For SCLC, polyethylene glycolated arginine deiminase (ADI-PEG20) and human recombinant polyethylene glycolated arginase (e.g., rhArgPEG, BCT-100, etc.) have been regarded as potential therapeutic targets due to arginine nutritional deficiency. And the combination of arginine with PD-1/PD-L1 inhibitors has also demonstrated efficacy in the clinic (76).

The combination of immunotherapy and metabolomics in lung cancer provides patients with more effective and personalized treatment options that are expected to improve their quality of life.

## 6 Discussion

In recent years, immunotherapies, particularly targeted therapies, have transformed the management and prognosis of lung cancer by providing personalized treatment options for lung cancer patients (86). And the integration of multi-omics data offers the unique advantage of aiming for a comprehensive assessment of each patient through extracted features, which promises a more complete picture of this complex immune ecosystem.

However, although ICI has been widely used in the treatment of lung cancer, there is still a lack of adequate understanding of prognostic biomarkers. We still need to increase research efforts on prognostic biomarkers for lung cancer at the multi-omics level such as proteomics and genomics. For example, genetic mutations in EGFR and KRAS have a key role in individualized therapy, but their specific impact and mechanisms in prognostic assessment need to be further explored (87, 88). In addition, proteomic markers such as CEA and CYFRA 21-1 show potential in disease surveillance, and their correlation with disease progression could provide additional information for disease management (89, 90).

Immune tolerance and therapeutic resistance are also current challenges in the combination of immunology and proteomics for the treatment of lung cancer. In particular, the EGFR T790M mutation leads to resistance to early drug (91–93). Specifically, although the third-generation EGFR-TKI ositinib has successfully treated patients with T790M mutations, new resistance mechanisms such as the EGFR T790M/C797S mutation are still emerging. Currently, there are investigators evaluating fourth-generation EGFR-TKIs clinically for new resistance issues (94, 95).

By integrating these multi-omics data, we can develop a more comprehensive understanding of the biological complexity of lung cancer, leading to the development of more effective therapeutic strategies and improved patient survival (96). Therefore, future research should focus on how to use these biomarkers to optimize treatment pathways, improve the accuracy of prognostic prediction, and ultimately achieve true precision medicine.
